# Passive acoustic sampling data of the Colección de Sonidos Ambientales *Mauricio Álvarez-Rebolledo* - Instituto Humboldt (IAvH-CSA) during 2018 and 2019 in Colombia

**DOI:** 10.1016/j.dib.2020.106648

**Published:** 2020-12-11

**Authors:** Orlando Acevedo-Charry, Daniela Murillo-Bedoya, Alexandra Buitrago-Cardona, Ana María Ospina-L, Claudia A. Medina-Uribe, Zuania Colón-Piñeiro, Bibiana Gómez-Valencia, Yenifer Herrera-Varón, Jose Manuel Ochoa-Quintero

**Affiliations:** a Instituto de Investigación de Recursos Biológicos Alexander von Humboldt, Carrera 8 # 15-08, Villa de Leyva, Boyacá, Colombia; b Asociación Colombiana de Ornitología, Bogotá, Colombia; c Reserva Natural Los Yátaros, Vereda La Caja, Gachantivá, Boyacá, Colombia; d Instituto de Investigación de Recursos Biológicos Alexander von Humboldt, Venado de Oro - Avenida Paseo Bolivar 16-20, Bogotá, D.C., Colombia

**Keywords:** Audible spectrum, Acoustic signals, National natural parks, Passive acoustic monitoring, Regional reserves

## Abstract

We present the dataset of passive acoustic sampling events deposited in the Colección de Sonidos Ambientales *Mauricio Álvarez-Rebolledo* at the Humboldt Institute (IAvH-CSA) during the years 2018–2019. The acoustic sampling events were generated from different projects, including Colombia Bio, Santander Bio, Boyacá Bio, Lisama, Riqueza Natural, and occasional events collected during this time. In total, 44,704 sampling events are deposited in the collection, corresponding to 1 minute of automatic recording sampled at a 44.1 kHz sampling rate and 16-bit resolution. The recording schedules correspond to 1 minute every 5, 10, or 30 min throughout the day, during 1 to 20 sampling days, across 79 localities in Colombia. The geographical coverage includes the departments of Bolívar, Boyacá, Caquetá, Cundinamarca, Meta, Santander, and Sucre. The present information was collected within the framework of the passive monitoring methodology established by the Humboldt Institute .

## Specifications Table

**Table:** 

Subject	Agricultural and Biological Sciences
Specific subject area	Ecology, Evolution, Behaviour and Systematics.
Type of data	Table Figures
How data were acquired	Field surveys
Data format	Table with raw data and figures
Parameters for data collection	Data considered corresponds to automatic recordings under passive acoustic monitoring samples, obtained from different places of Colombia. Here we provide geographic coordinates in decimal degrees. Each digital record is associated with a unique ID number to allow individual recognition and avoid duplicates. The dataset included collecting events from February 2018 to December 2019
Description of data collection	We collected digital records automatically by using acoustic sensors provided by the ARBIMON project of Sieve Analytics Inc, which were Motorola MotoG cellphone inside a protective case, with an external microphone attached, and the Arbimon Touch app for Android installed. We established a 1-minute recording within an interval of 5, 10, or 30 minutes of silence period, according to the sampling method for each sampling site.
Data source location	We obtained data from 79 localities across Colombia. We carried out the event samples in seven of 32 departments (22%): Bolívar, Boyacá, Caquetá, Cundinamarca, Meta, Santander, and Sucre. Our aim was to include part of the ecosystem, elevation and landscape gradient of Colombia [8], mainly in wilderness areas away of human settlements. Geographic coordinates presented in decimal degrees and reference datum WGS84: latitude: 0.56 and 10.029 min and max, respectively; longitude: −75.36 and −72.256, min and max, respectively. Elevation: 75 - 3425 m.
Data accessibility	Data available with this article and in [1]. https://ipt.biodiversidad.co/iavh/resource?r=sonidos_iavh-csa

## Value of the Data

• The dataset we present provides information on the acoustic print from several localities in Colombia between 2018 and 2019. Our contribution increases the acoustic knowledge of tropical biodiversity at the local and global levels; researchers often use these data to complement biodiversity studies.
• Information provided constitutes a basis for researchers interested in testing hypotheses related to species acoustic phenotypic traits or ecosystem acoustic signals. Decision-makers involved in environmental agendas, including protected areas management and land use planning strategies in Colombia, could also use our data.
• Passive sampling with acoustic sensors allows testing of questions about the different components of an acoustic system that characterize a particular ecosystem or species. Acoustic information can be potentially useful in developing conservation management initiatives [2], elucidating the effects of anthropic activities on the soundscapes [3], monitoring biodiversity (acoustic) responses in production systems [4]. Also, addressing basic research in species-specific processes by using novel methodologies like occupancy models [5] and automated identification to relate acoustic activity patterns with weather features [6].

## 1 Data Description

The dataset we report is a compilation of 44704 audio records obtained from passive sampling with acoustic sensors (Supplementary File 1). It includes digital specimens of 1 minute of recording, deposited in the Colección de Sonidos Ambientales *Mauricio Álvarez-Rebolledo* at the Humboldt Institute (IAvH-CSA). The access to this is in IPT of Instituto Humboldt [1], which is replicated in SIB Colombia (https://datos.biodiversidad.co/dataset/e98d3337-f7d7-454a-abae-c82d2d6010a9) and GBIF portals (https://www.gbif.org/dataset/e98d3337-f7d7-454a-abae-c82d2d6010a9).

**Fig. 1:**
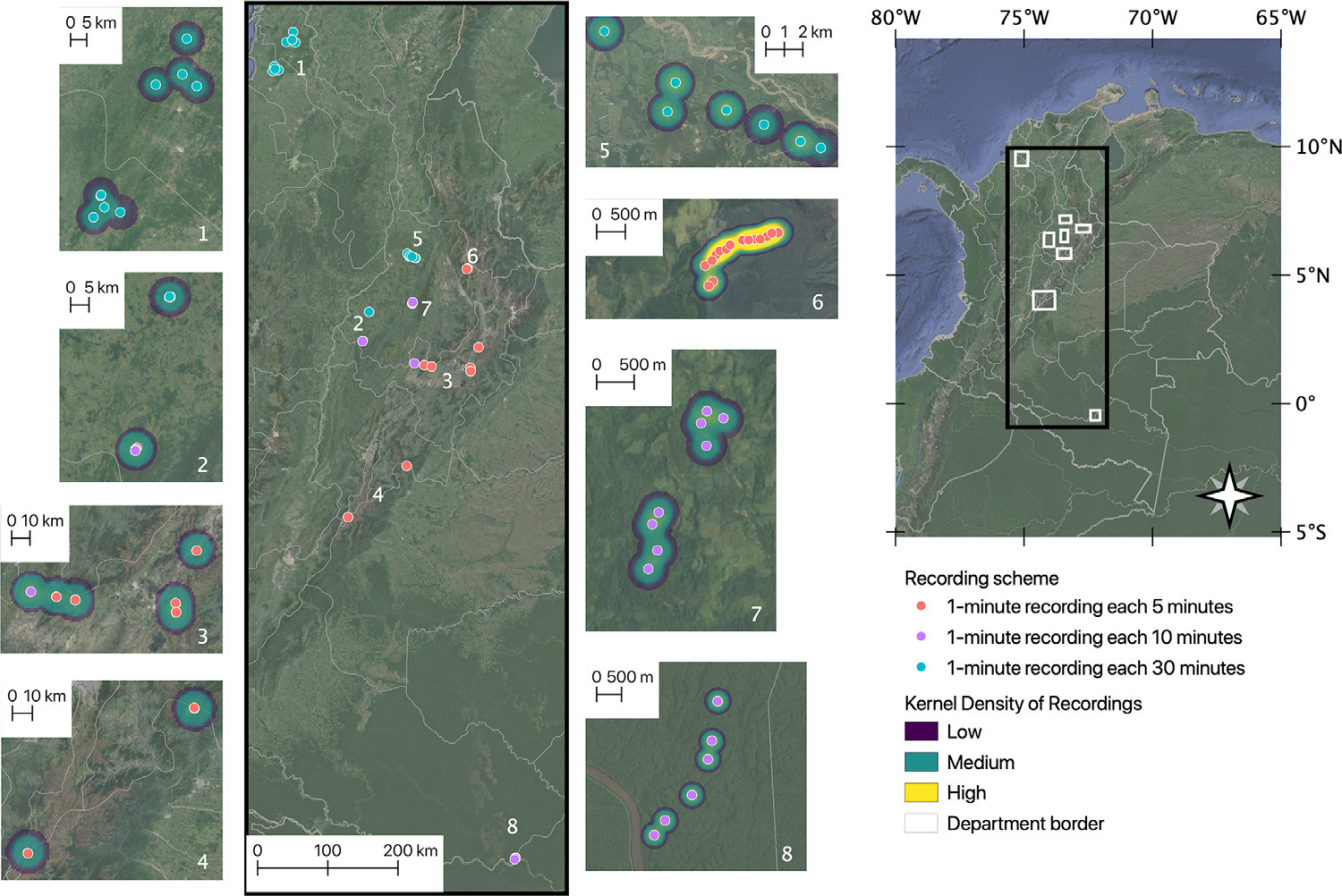
The geographical location of the 79 sites that include passive sampling with acoustic sensors by the Colección de Sonidos Ambientales *Mauricio Álvarez-Rebolledo*– Instituto Humboldt (IAvH-CSA) during 2018-2019. The 79 sites are presented in 8 selections on the map identified with white numbers. The color of the dot identifies the recording scheme. The halo around the points indicates the density of recordings, a number that changes regarding the scale. Right small-scale map includes the rectangles presented at the left maps.

**Fig. 2:**
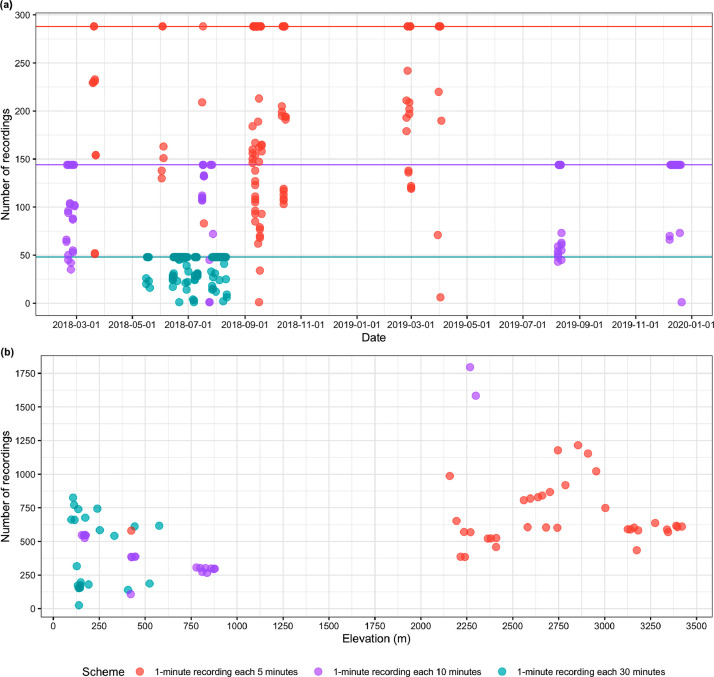
The number of recordings concerning date and elevation. (a) Each dot reflects the number of recordings per site per day, the horizontal lines represent the maximum number of recordings per day for each recording scheme (1 minute recording every 5 minutes: 288 recordings; every 10 minutes: 144; every 30 minutes: 48); Full days of recording are identified by matching dots with lines in the same color. (b) Each point reflects the number of recordings per site along an elevation gradient (see Fig.1 for the location of the 79 sampled sites).

Our dataset provides information on the acoustic print of 79 localities from northern, southern, and eastern Colombia (Fig. 1). We represent the geographical distribution of all sampling records in Fig. 1; grouped by selections determined by closeness in the map and marked by colours representing the density of records (halo) per site and the recording scheme. The recordings were mostly concentrated in the departments of Santander (21998) and Boyacá (12099) (Supplementary File 1). The recording scheme established for the recorder device was different among places (Fig. 2). We selected three schemes that vary in time of the silence period: 1 minute of record and 5, 10, or 30 minutes of not recording (Fig. 2). The number of records is presented per day (Fig. 2a) and in an elevational gradient (Fig 2b) and is discriminated using a different colour according to the recording scheme.

## 2 Experimental Design, Materials and Methods

### 2.1 Study area

We have records from 79 localities across Colombia (Fig. 1), corresponding to seven departments and representing different ecosystems. Our sites include specific ecosystems or protected areas representative of Colombian landscape: paramos of Sumapaz, El Valle, and Ocetá; dry forests of Montes de María and the middle Chicamocha river valley; buffer zone of Serranía de Los Yariguíes and Chingaza Natural National Parks [7]; Natural Reserve Los Yátaros; Muinane indigenous territory of Yarí River and Lizama stream. The municipalities location in the surveys are in three zones within the country, and they cover an elevational gradient from low to high areas. In the northern, San Jacinto and San Juan Nepomuceno, Bolívar (240–443 m); Chalán and Colosó, Sucre (174–576 m); in the eastern, Barrancabermeja, Bolívar, Cimitarra, El Carmen de Chucurí, San Vicente de Chucurí, and Santa Bárbara, Santander (98–3004 m); Arcabuco, Cómbita, Gachantivá, Monguí and Socha, Boyacá (2157–3420 m); San Juanito, Meta (2216–2270 m); Cabrera, Cundinamarca (3339–3345 m); and in the southern, Solano, Caquetá (155–176 m, accessing by Puerto Santander, Amazonas). We attempt to include a variety of ecosystems, environment, or landscapes gradients of Colombia.

### 2.2 Data collection

To collect data, we used automatic recorders assembled by the ARBIMON project of Sieve Analytics Inc [2,3], for the period between February 2018 and December 2019. The recorders consist of a Motorola MotoG cellphone inside a protective case, with an external microphone attached [2,3]. Recorders had installed the Arbimon Touch app for Android, which allows us to select different recording schemes. According to each project, we choose among three recording schemes of a 1 min sound varying the silence period to 5, 10, or 30 min. Researchers selected each sampling point by following its own criteria but trying to include at least 50 to 100 m of the same coverage to capture the acoustic habitat footprint and assuming independence of each point. Each site was as away as possible to human settlement. Although environmental barriers may influence this type of recorder, it captures sounds about 50 m around [5]. Recorders lasted in the field for periods of 1 to 20 days.

We verify each recorder operation by checking that time and date were correct, as well as the battery level at 100% and storage empty. We assigned a unique name to each sampling point to track each data collection event. We discarded three sites where the recorder was not correctly installed (date and time failed) within the quality control process. The final matrix obtained was verified by the Infraestructura Institucional de Datos (I2D) personal of the Humboldt Institute.

We provide an organized dataset with the metadata that constitutes a useful source of information, but the visualization and analysis are still a challenge of a dynamic in Colombian and Neotropical ecoacoustics [8]. We hope in the near future, the Colombian ecoacoustic community will have an interactive platform for storage, visualization, and modular analysis (e.g., [9]), that allows the integration of different approximations in this topic. Links presented for each file in our dataset may serve as a supply to create a platform with those characteristics.

## Ethics Statement

Some of the recordings could include voices of humans, the most likely source are the voices of the person that installed or removed the devices in the field. The probability of recording other human voices is very low given that we deployed each acoustic sensor in wilderness areas away of human settlements; we tried to preclude linking any recorded voices to individuals.

## Declaration of Competing Interest

No potential competing interests were declared by the authors.

## Appendix Supplementary materials

Image, application 1

## Footnotes

Supplementary material associated with this article can be found in the online version at doi:10.1016/j.dib.2020.106648.
